# Supplementary material for: Characterization of p53 Family Homologs in Evolutionary Remote Branches of Holozoa
Source: Int J Mol Sci. 2019 Dec 18;21(1):6. doi: 10.3390/ijms21010006 (PMC6981761; doi:10.3390/ijms21010006)
Supplement: Supplementary file 1 [file ijms-21-00006-s001.zip › Supplementary material 01.pdf]

Characterization of p53 family homologs in evolutionary remote branches of Holozoa

Václav Brázda, Martin Bartas, Jiří Červeň and Petr Pečinka

Supplementary material 01: All eleven non-metazoan p53 family homologous sequences in FASTA format

```
>XP_001746020.1_Monosiga_brevicollis
MASQDTLPASQETVPDAETGVFPPMLTRHGSSYKRMSMRDFHEY
LSRINKDGDMASGSMVRRDCMMHKDGWQQVDAEFDVDIDILGGQLPMDTELVDLETPL
PGTGLDDTPQASAKPCGALEDSGEPNPAGFRANLADSSVAAGPGARAIGWTYSPILNT
LFTPMDYSCPIRFATNESVPDLSRIVAHLEYTQTNQRNFVVNRCDMHRQGDSGPFAEH
VLRVNNPQANYHQRQERLAVSVPVASTRSGKVEQNELFEWHCLTSCAGGINRRKIRVV
FRLIDPDQNVLGVQHINVRVCACPVRDRRTHEKAHAKREQANDKRNVIASDPTPSKKP
KLKDHPISMALRKMPSVSTLCQIGSGFDHDQDVQYLAICGRANYEVALKVRDQLNQSA
GLGRVAASSVHLPRSEVTLIKQRVAEATNLDEFLRACNLQSLKRTLENLGYTDLPLIA
TLDAREIEELTLTKQDQRRLKLALVTLNETDKAQDPYLARRRETKLMRVTVRSTPGQA
QAGGAGPTSPFAAAS

>XP_001747656.1_Monosiga_brevicollis
MEMSLFDDVVDGEVSSSSSSSSSSSSSSSSSSSRFPPLATNMNA
LTMIDWATPILSPLLSFLDSIMTSVVPGSVADGNERGPGQFQVAVETITLQHSDSDLM
VDIPGYGTTAVKRAPMTERDFEMLPSSFRGPYDLQLDLIDENSLKPRNTSPWTVSTPG
LDLTLLQNGSAPIPISSNSLHVPCRWPHFDVLLMIQYSPQLGRFFVHVNADVVLKIVL
ARAPPKGTDLVFRLRYALPEHRKTRVETCVTHQQAGSHFFGAPHNHLMSINREHVTYD
TDSTGHHYARVALDQFPFTDNVYSVPLRFHCFSSCPGSIARRMMQLMVYLEHSEHILG
ITSVDCRCCACPGRDRLSAEKRQQRNSAKRHPAASGTKSKRTRRSGDNMALASSQESN
EGESSQPSEDLSIHILPIHGKTHYRIIKEMIEGMSSARILTPNQTAVTAKANLKLQQR
LREHSQSHSNPPNLELLQRELSTGVSESPSLHKASARAMAIKEEPSAFKADGPTSGNN
SAVEAGDQGSTVPTEMDDQQSGDTEHTPHLDLVPEADALYRNDWKFLQVGTARAHEPG
QARTW

>XP_004991396.1_Salpingoeca_rosetta
MQAGARREDSLGRDIKVDIEDLLSTREASTIGISSFDVPFDQLE
LDRTCDFLIETVQLNDGFEDPTPVVDLPGKGRVKLPRVPTTSAELEHTPSKYPGPFNF
QLQFIDDSGGKPVPRATPFTYSPQAQRLFVQMNHKVPFRFSISDRVPNGTWLRIRTRF
TQPEFRNRQD

>XP_004991397.1_Salpingoeca_rosetta
MEVTTLVTHRADGMHAYHLVRCDMPEAVYEKDATGHYSIRIPFD
RCAAQNGTHVFYIELLCFSSCPGGLNRRPFEVTVSLEHSSRILGCDSIDTRVCACPGR
DRGAAEKRLRGDIGKKRGRKKKKPPTEPTVSMQDDNKIHTLLVQGRTHYAILHHLVEG
LDATVASSSSPPPSSSSSAAAAVLSSSAARNQAPSADKPLSEASFPAGNDAEETPGGD
TEPESEADDDNDEEEDEQQQLTPRQQVKYAFLNLRLQEAVRAWLSALRLEAYADMFMS
KGYDDLDVVCDLTQDDLDCLGISRPGHRKKLLLAAQKLKSLLHQARAVEAVPDRRLHK
TLSISRTTYRQALPSAD

>XP_004994590.1_Salpingoeca_rosetta
MSGHARLSRQASFANLLRGSSTESISTWISSLGRAPSLTRCSSS
FIRHVTPDASAPLSWAESTTNIEIAASDVPGFSEEFLNLTRSPTRTGLVAAQQQERQQ
QQQQEGEPQHYQQHHHQQQQQQQQQQQQQQQHHEQQSSALTLNLNMEADTRGSAKASS
WMYSGTLHQLFTNIDQVFPVQITSSESVPESAKLHVQLQFSKDPSHPIPRCPTHLAED
TAPHAEHVLQTTMPTADYHMDTSGRAYLVVPIERSKDEGLLCQTLLLKMRCFSSCSGG
MNRRPVDIVFTLINGAQLLAQVSVAVRVCACPARDCRNMERQQAKRAAKSHQGKDVRH
PAKKGKVRGITAICKRQDADLRDSDTFSLNIKGRANYHIIRRIAEALKTNQVNVPWNR
SAGAIVIPDNIHDVASVPEFLARLGLTQYQPLFMGSGFDDLEVLEHLDLNDLSAIGVE
DGIHQDTLLYAAAKLARSQQHHGEGCRPRGRCTSLHSPDVVDTHDHNNSTISISSNCH
VFPDTSQQMQYTTQPHFPPMQLAPSNGVPHSLPSAAPSVAHAAKPSSGPGTSTSSAVA
TTAPVQQQQQQQQQQQQQQQQQQQQQQQQQQHSGPLIHLPVPSSSSA

>XP_004365382.2_Capsaspora_owczarzaki
MDMVRLTRGQSFQQQQQQQKSASQQNASGDGAAAAGAGAGAGAGAAAGADADADSKGSHDPADLNSTLLL
GDSQFGETTGTSLLNMSGLRGFNTPSSSSSHHSHHSHHSNSSNSSFLGRPRTLPVQKPLSILQGSAPEPF
```

VEPLTIGEPIYASPNSLGEAGFLLSVDVSNARHSAISSAYSYSEALGTLFTNFDVGVPFVFRVAKPPPTV
HPLHIRATLRYKQMQFMKEPVRRCPLHLSIDSDLHLLRACDQDTVYSVDYHGRASIAVPFTPTMQPLVPV
LINTLKDAHVPLVTRHPSSTHGEASRSNPYVCWSMTWFLKFMCYTSCTGGMNRRATEIVFTLEDSQGLIY
GAQALDFRTCASPSRDRKQLEKSPPPIPVGSIIPPEVVTAHQGSALADPGTTASTPTRRSAQPVVPSPVV
AASPTPAPGLLSSGRKAGAIPVTGVTPRPLVVIPAASSAQTAPTAPAAAIAAPSPALTEAHAAPTTLLRD
RSITAIAKRRHADDDDEIFTISIRGRDNFVLVSKIVEGLNALAQRTSAAVASKLERTTTPPVVEDDPSAP
PALSALDTLAQAAAGHVRAPDEAAPATHERKRARLADDDTKAGRATEASGESISTWLGSFGLKQYAVNFA
KNGYTNTPQILNLCEDDLKSIVRVKAHRVRIAAAIDELADAQAEPTSTPPMSSDGDVTVVRMTIRRTTSS
TAK

>XP_014156832.1_Sphaeroforma_arctica
MYNLPLQDHAPAAEPSTSDPQLNIQPELTLPPPRQTPPVLDSSHTPMPGIYNFRVRLGHFDSTNPTGKLT
GSTTYSSLLDRLFINMGVYTPITLFSHTPPPEGAVVRCFVVFSSQEKCTTTVTRCPNHRSATDHSNTGND
PQKAPLTHILQVSHPQAQYLTTPSGVESAVVPYNARESSVKNSAAKKERTAGFGAFQDTTSIRYMCYSSC
AGSINRENIHTVFRLELGSDILGVASLDTKICSCPSRDRTKAEKLTRQQEASGTTGSSRKRSEKGRDSPS
HSTDIASTGATKDSHTSERIQAPSIVHPHSSQATLVGKRSQRLIQNVQHKLPTRINTADTHTAHPMRWST
AQSTRIATADSTVGIDEDAVYYVAVRGRQNYEVLSRVADGLNALAQRPPRARSFRQHNPLPTRTPSLLVT
SNPPPPPTTTANTAAPTNTNTNTNTNTSTDTKTVFSRPGQNSTIPSLSSRRNPSLVSSTTSVPPAQPRTA
ASSRSLRTVPSRERRVSGTVPSDVPVSESPARNTR

>CFRG4869T1_Creolimax_fragrantissima
MTESESADMTGKLGQPSMSFSQIFNSFAENGSTVVGEHGNEVGQEVMFSVMRRSTSNAQATMATRDYNTNAQQTFQPDG
QATNAGYYASEPLEDNGLYSGRIEIEDGGYQYIMNPMNNPLGILPATLPSYLDNVSASYDAHGSLANLIGLPFVNDGRA
ADSNAQGIQSHPELVPPAAQSPPLPLDSSHTPVPGIYGFGVNVGHLDTSNPTGKLNGSTTYSKLLDRLFININVYTPIT
IATNKQPPPGAMVRCFVVFSSQEKCRDIVTRCPNHRSPTDPTNQSVDANEVPLSHMLHVSHPKALYCPSNTGIESVLIP
YRPTEISFAKASKIKRPSGTTDSTTDAPTLQEHTYVRFMCFSSCPGSINRENIHAVFRLELGDEILGVASLDTKICSCP
SRDKAKAEKLARAHVGMSDRNSNAKSMKHEVRGSDGAGTVKRRSPITHSVSSASTPSNQKGFVGPASVLTRSASQRLQE
NNMQTRTKGQADSSMYKSNIHSHPNGININGSTKLMARGREDSNRLGSGVDNGLEHTNGGGSTRTYYNVSAMLNSNTRE
PGGHNKFDDELYYVTVRGRKNYEILSRVAEGLTCTSQKPQRALSYRRHPGAPAKVQSIVVSNHSSKEPVKPISSERARA
TAIPRVVTSSITSPHIPTSQGDITTPKRSKRLLVSSEAKIQSPARRAIGKGVDITVNGSEVRQNNPKKSRVNGQNKPAT
RQGNKTHRAVQNNADGSSTEEEPELADRSIIPPLTSKSRISVPTRSHQTRRSSARLN

>Ihof_evm3s137_Ichthyophonus_hoferi
MHNLHNLLPRQNTSALGTKTKDNESNRTGRENQLQHDTLELSLERMDVDIDLSNNMFGLPSFPNSGLTNY
DLTSTTNSVVDMLEYLDPILTPSPVLDPSWALPQAQHQAQPQSQQLPLPMLTRSQSNARFEEPNFEGPYH
FNISFVTKTESGGKSDAKGATWLYGSQRDLLYVTMNTTVPINLTIEGNPPADANVRVKVVYANPEDYMER
VVRCPTHADRKEQATKPFPDQIIRVDHLAATNELDRLNHYSVLIPYSKLKSRPQLRLRYLCYSSCVASIN
KRPVSTVIILEDASGRLLGQRSVSTKICACPGRDLKKDNPITAEKDKWKNNRNVKGQQTNDIDPHPSRTT
FVNPKNEILQSTKSIVQDNNNMYTVNRTIRGRANFEMINKIIDSLNLMDNLQNDSINPNHSSVDPPALHR
QFNLRDMCPTLALIPPPIIQNHKTKEQRMVSREKTPTIESDEEEIVRDTKPTKKFKQGSPFSPPGPSLQK
TNSFSRMQ

>Nk52_evm78s1737_Chromosphaera_perkinsii
MSFPNQNRVPGSEQTWLENLVMRFPSEGGGNEFMLNNRHHHHPNKENEMLDEVEFKFEAVNCFSAVPDTF
QAGGSSNSNMSGVGDKDSSCHSDSVDRYIQGHLQPVGNHQSNSNNNNRCRAPLVPTNSLGRESTSSNTTA
PEILGRNYVRGALSYEFPHNVKGESGAHDRKLSLDAGRTAAMDSASRSSPLLQSPKAELSSLVDENALQH
PPETSNISTCPSERGFARLEQVLDDVAVDGTSAQHSDVYDGNAQVREQIPFDVCFEGSSLQSKATTWGYS
STLNKVFLNLDTLCPIPLSIEQENIPRGFILRATARFTKPQHYNETVVRCPNHEQKDPNFKSSHKDKIVP
TSHVVRCENPSAVYETDAASGHHSVYIPIHTRTKSGSSDLSSFADDSKANDNGNDDKPKKTNIIVPFKFM
CFSSCPGGINRRPFELQLTIENNGVVFATRSVGIRVCACPLRDKRSEELTFMQKNKRLLVDCEKEEESTG
AHGSDEKSSKWLVSKRRKTNIPSSLSGNSLASLSKTVPSTIDKHGNEIFNIKVKGRQNYDIVARVVEGLN
ALYGDITHGRQKNAEKKAIGSKPGDYSGTGTDTAEESDEEESEMSGRKGQEDKLMTSYLEVLSKYSPEAL
KCMPVREFLKAVNLDTYYQSFKDNGYDDIQLVTCIDKSDLDILNISLGGHRKKLVAAARALFSRLKGPVE
TTRRRGSIVYTQTTKRGSFTMKKTYSATSSQPSGSIFPADFPNIISSLGGKDDGSL

>Clim_evm153s157_Corallochytrium_limacisporum
MSFQQRGPMKAPPPPLDLQGSGANRPELMRQQSQDGAERAITALAQVAFQESVALVEGRIQFVDRNQVLN
QQTNGGPGNANGEEAKPQSTEPSKDSSALLLDVPMADIGLLLNKEQEEELKKEVKRKVTYFDVDIPTHKP
FGAFSKKLEKLFALPHVPYLFSFYTPITEGSIQIKLKFKNPYYQHEPVERCEQCSKKEEPQPDAPKHFVT
CTADERGESNAEHTITDDGPIVTMRFNLQREVAPRVNTGSGNVHCVQRNLKFGCLNSHLGGKNKRDLEIN
LSLLSTEGVQLASKTIDLKVCKRPQRDADLEEQGEAMESVDAMGMYGNVPQGQRPPQEEKDKSKEDWHLI

KVRDFEDWKLLNQMAEALSFFREHVHNAYSQKLLSGQKGEDQDIGSKRGPPDADGPAAKLRKLSPEENQR
TLYEWLERNGLKQLQPVLASNNVHSFDDLWSICASEQVPFLPGDVKQKMKTILEKDNPRADAVRDAFENA
TRLLLKMEITKVFDLNDSQGNAFKGLQRNAVPTPAAPLSAILPSPARFTTQNADWLLQAGSLDELNVRFA
NPFSPLITPGLQAELLSGQNHFFGQNSLDMIHQQQQTNERLGIGLNPTHVQQHQEQPTPDIFF
