# Supplementary material for: Characterization of p53 Family Homologs in Evolutionary Remote Branches of Holozoa
Source: Int J Mol Sci. 2019 Dec 18;21(1):6. doi: 10.3390/ijms21010006 (PMC6981761; doi:10.3390/ijms21010006)

Characterization of p53 family homologs in evolutionary remote branches of Holozoa

Václav Brázda, Martin Bartas, Jiří Červeň and Petr Pečinka

Supplementary material 06: Detailed DNA tree (based on CDS sequences) of the most distant p53 homologs, related to Figure 4.

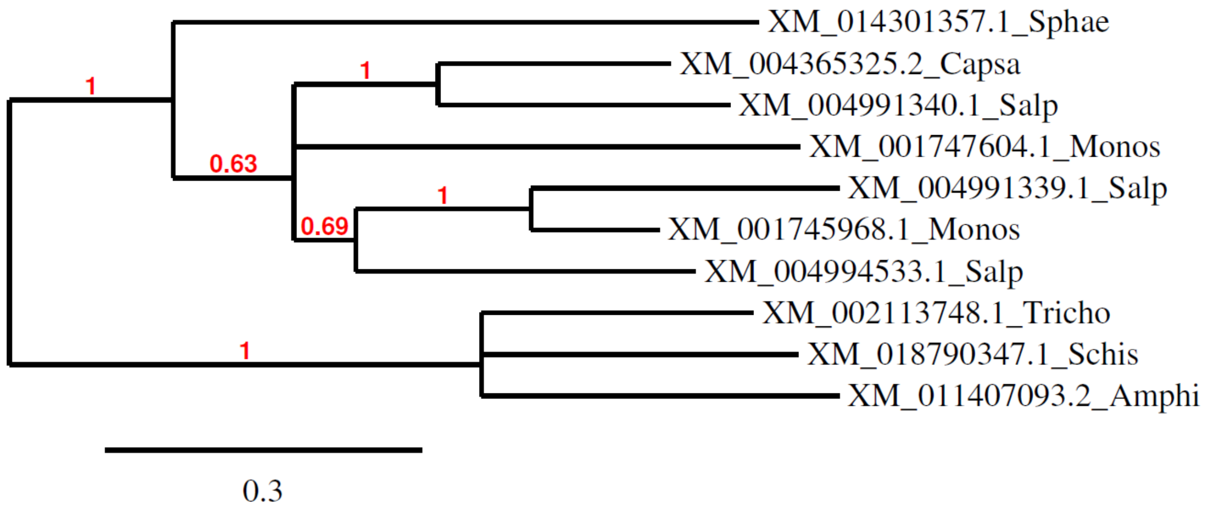

Supplement: Supplementary file 1 [file ijms-21-00006-s001.zip › Supplementary material 06 Detailed DNA tree (based on CDS sequences) of the most distant p53 homologs, related to Figure 4..pdf]
