# Supplementary material for: Characterization of p53 Family Homologs in Evolutionary Remote Branches of Holozoa
Source: Int J Mol Sci. 2019 Dec 18;21(1):6. doi: 10.3390/ijms21010006 (PMC6981761; doi:10.3390/ijms21010006)
Supplement: Supplementary file 1 [file ijms-21-00006-s001.zip › Supplementary material 09 Estimation of evolutionary age.pdf]

Characterization of p53 family homologs in evolutionary remote branches of Holozoa					
Václav Brázda, Martin Bartas, Jiří Červeň and Petr Pečinka					
**Supplementary material 9. Evolutionary age of particular phylogenetic nodes was mined from TIMETREE database (http://www.timetree.org/)**					
**taxon A**	**taxon B**	**node name**	**estimated time (MYA)**	**lower CI (MYA)**	**upper CI (MYA)**
Homo sapiens	*Mus musculus*	Euarchontoglires	90	85	94
Homo sapiens	*Danio rerio*	Euteleostomi	435	425	446
Homo sapiens	*Callorhinchus milii*	Gnathostomata	473	450	497
Homo sapiens	*Branchiostoma floridae*	Chordata	684	598	787
Drosophila melanogaster	*Caenorhabditis elegans*	unknown	743	551	946
Drosophila melanogaster	*Mya arenaria*	Protostomia	753	642	864
Homo sapiens	*Nematostella vectensis*	unknown	824	611	1035
Homo sapiens	*Amphimedon queenslandica*	Metazoa	952	757	1147
Homo sapiens	Holozoa group	unknown	1023	691	1354
Homo sapiens	*Entamoeba histolytica*	unknown	1480	1082	1781
