# Supplementary material for: Characterization of p53 Family Homologs in Evolutionary Remote Branches of Holozoa
Source: Int J Mol Sci. 2019 Dec 18;21(1):6. doi: 10.3390/ijms21010006 (PMC6981761; doi:10.3390/ijms21010006)
Supplement: Supplementary file 1 [file ijms-21-00006-s001.zip › Supplementary material 07 Phmmer of putative DBD from Entamoeba histolytica.pdf]

**Characterization of p53 family homologs in evolutionary remote branches of Holozoa**

Václav Brázda, Martin Bartas, Jiří Červeň and Petr Pečinka

**Supplementary material 7: Phmmer of the putative p53 family DBD sequence from *Entamoeba histolytica* (Q868M8). Results with E-value lower than 1 are reported. The red line depicts E-value lower than 0.01. The analysis identifies only the sequence itself and two mutant versions of the same protein, compared to the thousands of significant hits including human p53, p63 and p73 for our novel characterized p53 family homologs in Supplementary material 2. The lower part of the table shows the most significant domain found in *E. histolytica* Q868M8 by SMART, which is highly similar to the alpha kinase Pfam domain. No similarities with the p53 family DBD by contemporary validated methods were identified.**

| **Phmmer of putative DBD from *Entamoeba histolytica* (Reference Proteomes database)** | | | |
|---|---|---|---|
| **Target** | **Description** | **Species** | **E-value** |
| Q868M8_ENTHI | *Q868M8, annotated as "p53-like protein"* | *Entamoeba histolytica* | 5.30E-129 |
| B1N3R5_ENTHI | *Uncharacterized protein (mutant of Q868M8))* | *Entamoeba histolytica* | 8.30E-129 |
| C4MBK2_ENTHI | *p53-like protein (mutant of Q868M8)* | *Entamoeba histolytica* | 4.00E-128 |
| A0A0N5AEU6_9BILA | Uncharacterized protein | *Syphacia muris* | 0.075 |
| A0A080K1V4_9NEIS | Transcriptional regulator | *Snodgrassella alvi* SCGC AB-598-O02 | 0.083 |
| C4LWZ0_ENTHI | Elongation factor 2 kinase putative | *Entamoeba histolytica* | 0.11 |
| A0A0A1UFE6_ENTIV | Elongation factor 2 kinase, putative | *Entamoeba invadens* IP1 | 0.19 |
| A0A183EY03_9BILA | Uncharacterized protein | *Gongylonema pulchrum* | 0.46 |

| **The most significant Pfam domain found in *Entamoeba histolytica* (Q868M8) by SMART** | | |
|---|---|---|
| 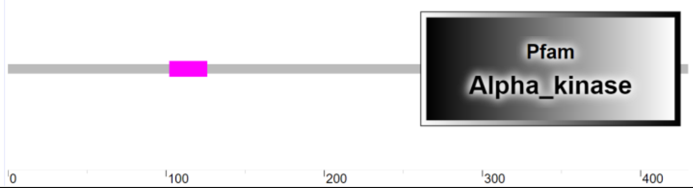 | **Domain** | **E-value** |
| | Low_complexity (102 – 126 aa) | NA |
| | Pfam: alpha_kinase (261 – 425 aa) | 2.5E-8 |
