# Supplementary material for: Characterization of p53 Family Homologs in Evolutionary Remote Branches of Holozoa
Source: Int J Mol Sci. 2019 Dec 18;21(1):6. doi: 10.3390/ijms21010006 (PMC6981761; doi:10.3390/ijms21010006)
Supplement: Supplementary file 1 [file ijms-21-00006-s001.zip › Supplementary material 08 p-values of all domain hits in non-metazoan p53 homologs.pdf]

Characterization of p53 family homologs in evolutionary remote branches of Holozoa

Václav Brázda, Martin Bartas, Jiří Červeň and Petr Pečinka

Supplementary material 8: p-values of all domain hits in non-metazoan p53 homologs

Query	Hit type	PSSM-ID	From	To	E-Value	Bitscore	Accession	Short name	Incomplete	Superfamily
Q#1 - >XP_001746020.1_Monosiga_brevicollis	specific	176262	132	311	5.49E-71	224.07	cd08367	P53	-	cl14608
Q#2 - >XP_001747656.1_Monosiga_brevicollis	specific	176262	194	357	5.83E-58	191.328	cd08367	P53	-	cl14608
Q#3 - >XP_004991396.1_Salpingoeca_rosetta	superfamily	326329	114	167	1.71E-12	61.9012	cl14608	P53 superfamily	C	-
Q#4 - >XP_004991397.1_Salpingoeca_rosetta	superfamily	326329	3	110	9.41E-28	106.584	cl14608	P53 superfamily	N	-
Q#4 - >XP_004991397.1_Salpingoeca_rosetta	specific	334133	256	315	2.08E-16	72.2856	pfam00536	SAM_1	-	cl15755
Q#5 - >XP_004994590.1_Salpingoeca_rosetta	specific	176262	151	319	1.16E-40	145.875	cd08367	P53	-	cl14608
Q#5 - >XP_004994590.1_Salpingoeca_rosetta	specific	336753	404	465	1.48E-09	54.1979	pfam07647	SAM_2	-	cl15755
Q#5 - >XP_004994590.1_Salpingoeca_rosetta	superfamily	311579	357	381	0.01354	33.7967	cl06653	P53_tetramer superfamily	C	-
Q#6 - >XP_004365382.2_Capsaspora_owczarzaki	specific	176262	184	373	3.98E-40	145.104	cd08367	P53	-	cl14608
Q#6 - >XP_004365382.2_Capsaspora_owczarzaki	superfamily	353976	612	670	2.00E-08	51.3751	cl15755	SAM_superfamily superfamily	-	-
Q#6 - >XP_004365382.2_Capsaspora_owczarzaki	superfamily	311579	500	529	3.24E-05	41.1155	cl06653	P53_tetramer superfamily	C	-
Q#6 - >XP_004365382.2_Capsaspora_owczarzaki	superfamily	225689	362	449	0.000544872	41.7963	cl34559	DedD superfamily	C	-
Q#6 - >XP_004365382.2_Capsaspora_owczarzaki	superfamily	235906	398	609	0.00724794	39.4476	cl35530	PRK07003 superfamily	NC	-
Q#7 - >XP_014156832.1_Sphaeroforma_arctica	specific	176262	53	254	4.50E-44	153.579	cd08367	P53	-	cl14608
Q#7 - >XP_014156832.1_Sphaeroforma_arctica	superfamily	311579	366	391	0.000771266	36.8783	cl06653	P53_tetramer superfamily	C	-
Q#8 - >CFRG4869T1_Creolimax_fragrantissima	specific	176262	209	404	5.08E-43	153.579	cd08367	P53	-	cl14608
Q#8 - >CFRG4869T1_Creolimax_fragrantissima	superfamily	311579	561	585	4.08E-06	43.8119	cl06653	P53_tetramer superfamily	C	-
Q#9 - >Ihof_evm3s137_Ichthyophonus_hoferi	superfamily	326329	141	318	9.47E-41	144.334	cl14608	P53 superfamily	-	-
Q#9 - >Ihof_evm3s137_Ichthyophonus_hoferi	superfamily	311579	366	399	0.000175661	38.8043	cl06653	P53_tetramer superfamily	-	-
Q#10 - >Nk52_evm78s1737_Chromosphaera_perkinsii	specific	176262	261	468	8.67E-53	180.543	cd08367	P53	-	cl14608
Q#10 - >Nk52_evm78s1737_Chromosphaera_perkinsii	superfamily	353976	635	691	7.89E-10	55.1444	cl15755	SAM_superfamily superfamily	-	-
Q#11 - >Clim_evm153s157_Corallochytrium_limacisporum	superfamily	326329	131	313	3.99E-15	73.4572	cl14608	P53 superfamily	-	-
