# Supplementary material for: Characterization of p53 Family Homologs in Evolutionary Remote Branches of Holozoa
Source: Int J Mol Sci. 2019 Dec 18;21(1):6. doi: 10.3390/ijms21010006 (PMC6981761; doi:10.3390/ijms21010006)
Supplement: Supplementary file 1 [file ijms-21-00006-s001.zip › Supplementary material 10 Entamoeba histolytica putative p53 homolog DBD sequence.pdf]

Characterization of p53 family homologs in evolutionary remote branches of Holozoa

Václav Brázda, Martin Bartas, Jiří Červeň and Petr Pečinka

Supplementary material 10: Entamoeba histolytica putative p53 homolog DBD sequence

>entamoeba_histolytica_putative_p53_Q868M8_DBD

RKMVKKQEREEDNKDIYNIIEMINKKEIINKMIIHISSSINKERKEYNQKEIIQEFIYSTNDYCIVNLKRNNENIINDLMKI
RMNDMFSKYLPTKPFSCIVFKKSSLYQIIPFSNKLNGYIITIPQNYFYNQNCWEFYERPPFYLTTPATILLSSNYLIGNNL
FISKCECPIININHFFAFKERRLSYNSDILWAINTIEKQ
