# Supplementary material for: Characterization of p53 Family Homologs in Evolutionary Remote Branches of Holozoa
Source: Int J Mol Sci. 2019 Dec 18;21(1):6. doi: 10.3390/ijms21010006 (PMC6981761; doi:10.3390/ijms21010006)

Characterization of p53 family homologs in evolutionary remote branches of Holozoa

Václav Brázda, Martin Bartas, Jiří Červeň and Petr Pečinka

Supplementary material 5: Whole alignment of primary amino acid DBD sequences of the most evolutionarily distant homologs of p53 family compared to human p53 with depicted conserved regions II V

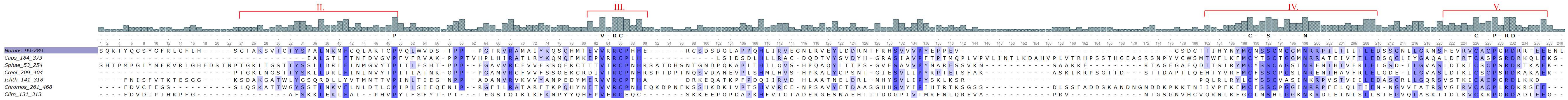

Supplement: Supplementary file 1 [file ijms-21-00006-s001.zip › Supplementary material 05 Whole alignment .pdf]
