# Supplementary material for: Characterization of p53 Family Homologs in Evolutionary Remote Branches of Holozoa
Source: Int J Mol Sci. 2019 Dec 18;21(1):6. doi: 10.3390/ijms21010006 (PMC6981761; doi:10.3390/ijms21010006)
Supplement: Supplementary file 1 [file ijms-21-00006-s001.zip › Supplementary material 11 DBDs sequences of the most evolutionarily distant homologs of p53 family for alignment with human p53 DBD.pdf]

Characterization of p53 family homologs in evolutionary remote branches of Holozoa

Václav Brázda, Martin Bartas, Jiří Červeň and Petr Pečinka

Supplementary material 11: DBDs sequences of the most evolutionarily distant homologs of p53 family for alignment with human p53 DBD

>Caps_184_373

EALGTLFTNFDVGVPFVFRVAKPPPTVHPLHIRATLRYKQMQFMKEPVRRCPLHLSIDSDLHLLRACDQDTVYSVDY
HGRASIAVPFTPTMQPLVPVLINTLKDAHVPLVTRHPSSTHGEASRSNPYVCWSMTWFLKFMCYTSCTGG
MNRRATEIVFTLEDSQGLIYGAQALDFRTCASPSRDRKQLEKS

>Sphae_53_254

SHTPMPGIYNFRVRLGHFDSTNPTGKLTGSTTYSSLLDRLFINMGVYTPITLFSHTPPPEGAVVRCFVVFSSQEKCTTT
VTRCPNHRSATDHSNTGND
PQKAPLTHILQVSHPQAQYLTTPSGVESAVVPYNARESSVKNSAAKKERTAGFGAFQDTTSIRYMCYSSCAGSINRE
NIHTVFRLELGSDILGVASLDTKICSCPSRDRTKAEK

>Creol_209_404

PTGKLNGSTTYSKLLDRLFININVYTPITIATNKQPPPGAMVRCFVVFSSQEKCRDIVTRCPNHRSPTDPTNQSVDAN
EVPLSHMLHVSHPKALYCPSNTGIESVLIPYRPTEISFAKASKIKRPSGTTDSTTDAPTLQEHTYVRFMCFSSCPGSINR
ENIHAVFRLELGDEILGVASLDTKICSCPSRDKAKAEK

>Ichth_141_318

FNISFVTKTESGGKSDAKGATWLYGSQRDLLYVTMNTTVPINLTIEGNPPADANVRVKVVYANPEDYMERVVRCPT
HADRKEQATKPFPDQIIRVDHLAATNELDRLNHYSVLIPYSKLKSRPQLRLRYLCYSSCVASINKRPVSTVIILEDASGRL
LGQRSVSTKICACPGRDLKKD

>Chromos_261_468

FDVCFEGSSLQSKATTWGYSSTLNKVFLNLDTLCPIPLSIEQENIPRGFILRATARFTKPQHYNETVVRCPNHEQKDPN
FKSSHKDKIVPTSHVVRCENPSAVYETDAASGHHSVYIPIHTRTKSGSSDLSSFADDSKANDNGNDDKPKKTNIIVPF
KFMCFSSCPGGINRRPFELQLTIENNGVVFATRSVGIRVCACPLRDKRSEE

>Clim_131_313

FDVDIPTHKPFGAFSKKLEKLFALPHVPYLFSFYTPITEGSIQIKLKFKNPYYQHEPVERCEQCSKKEEPQPDAPKHFVT
CTADERGESNAEHTITDDGPIVTMRFNLQREVAPRVNTGSGNVHCVQRNLKFGCLNSHLGGKNKRDLEINLSLLST
EGVQLASKTIDLKVCKRPQRDADLEEQ

>Homos_99-289

SQKTYQGSYGFRLGFLHSGTAKSVTCTYSPALNKMFCQLAKTCPVQLWVDSTPPPGTRVRAMAIYKQSQHMTEVV
RRCPHHERCSDSDGLAPPQHLIRVEGNLRVEYLDDRNTFRHSVVVPYEPPEVGSDCTTIHYNYMCNSSCMGGMNR
RPILTIITLEDSSGNLLGRNSFEVRVCACPGRDRRTEEENL
