# Supplementary material for: Characterization of p53 Family Homologs in Evolutionary Remote Branches of Holozoa
Source: Int J Mol Sci. 2019 Dec 18;21(1):6. doi: 10.3390/ijms21010006 (PMC6981761; doi:10.3390/ijms21010006)
Supplement: Supplementary file 1 [file ijms-21-00006-s001.zip › Supplementary material 12 FASTA sequences of eleven non-Metazoan p53 family homologous sequences and twenty-seven other representative closest relative Metazoan species for protein tree construction.pdf]

Characterization of p53 family homologs in evolutionary remote branches of Holozoa

Václav Brázda, Martin Bartas, Jiří Červeň and Petr Pečinka

Supplementary material 12: FASTA sequences of eleven non-Metazoan p53 family homologous sequences and twenty-seven other representative closest relative Metazoan species for protein tree construction

>XP_020604944.1 cellular tumor antigen p53-like isoform X1 [Orbicella faveolata]
MFLPQNPSTSPPADPPSTAVSRDSNVMVTCGPKDRPGYTFTGEAFRKILTNPDLNKMADQVEPTIGPLINEKQLKQ
LMEDVRGSDDGNCFLSMGGGEDSQDLSFGVQSDMRGYGPQYHYISSSQAVHDLPTSSSPQPPAYPHNIPYSPGP
VAGHRSSSTCISPISTTPYETCSNYSEASTSGPSTPPPGLTSSYMPIHARLPTIPSTTEYPGNWNFQISFGPLTESASKSA
TWTYSDVCKKLYVNLASFCPIKFKTNSPPPPGTYLRAVAVFKGSTNLHDIVKRCPNHMESSNDGSAPNNHFIRSNNP
AARYHTCPESSRHSVLLPYNGPQVGTEYVTEMFAFMCFSSCASGPSRRPVEVIFTLEQNGQTLGRRVVEIRVCACPG
RDRKSDEKVACGEPSSSQGVRRTRKGSGNKGPSSKRRRTSSRSQDEEFIITTKNRHVYDILLEMKDSLEGKFESLNIQS
PEHSLVLSTSEDEADQPSTSGSPGAVFKRVGLSSM

>ABF72158.1 transcription factor p63 [Nematostella vectensis]
MSQSSSQESQILSQGTYEVLRSFVAEAEGAEYQYNVAPYSVDLTSCNEQENLEINIEKVALIPSDFLDYQGVNGFHTE
PVESSREQYHHHVPSAITPPPDYPTTNLVPANTDFPGEYGFDVGFDKENGPTPKSAPWTYSHQLQKLLCRMKCLVP
VRLVFRSKVPPEGFYIRAVVVYKQPEHFREVVERCANHITRQDDGHTAPKHLLRCENTKTLYRTCNLTGRHELMFPT
RKPDAGMDYFKDMFQFMCFNSCPGGLNRRPIIVIFTLELSGVVYGRKVLDVRVCACPGRDKADQESLETRKKNKM
KNLNTPPPTPDENKPSTSGETPAGSHNGVKEELESSHKSSSDKRDIRQCTKKGFELEHISKRIKREKEDEEEFTITVRGR
DNYEMLLKIKESLDLMNMVPQSVVDNYRKTCPPCGPTWFLDQQKPKPIQQVESSTDSELECSPPNSLSQLSRTSSN
ASSQGRRAAAEYSAVRFSLKRTVTYPPSRH

>XP_015761542.1 PREDICTED: cellular tumor antigen p53-like isoform X1 [Acropora digitifera]
MGVSVLCDFVFRVGIHVTCRGNHKTCQQRTRAAEGIRSFSFTREVNMAEEQVEPTISLNEKQFKHLLADVRGENSS
SCFLSMGEDSHDSLNFGQSDMRPFTQQFHSYVSSSQSPTSTSPQPSYSPQPPAYPQNIPASYSPTSNPANRTSISSSP
YDNCNTYNDGSATASSCSSQGIYMHARLPTIPSTTEYPGDWGFELSFGPLTESASKSATWTYSDVCKKLYVNLASFC
PIKFKTRMQPPAGTVLRAVAVFKGSTNLHDVVKRCPNHMESSNDGSAPKDQFIRSNNPAAQYHTCPESMRHSVLL
PYNGPQVGTEFVTEMFAFMCFSSCASGPSRRPVEVIFTLERDGQTLGRRVVEIRVCACPGRDRKSDEKAVSGEPTSS
QGIVKRARRGSGNKGPGAKRRRTSSRTSSEEEYVITTRNRHVYELLMDFKEKLEDKFEYVHQQNLDNSLVLSTSEDET
EQPSTSSVAPGSVFKRGGLSTM

>RMX37105.1 hypothetical protein pdam_00016598 [Pocillopora damicornis]
MVDNVEPTINTLLHDKHYKHLLDDVRGDENPSCFLSPLETGEDSQDNFLPSDMRSYGQPFPYIPSSQAPVDLPTSTS
PNPPAYPHNIPQSPGLVSVHRNSSSISPINTAHYETSSTYSDVSTPGTPATSSPNMVHTFMHPSRLPQIPSTTEYPGEL
NFQLSFGPLTESASKSATWTYSDVCKKLYVNLNSYCPIKFKTSCPPLPGTYLRAVAVFKGSTNLHDVVKRCPNHMEN
SNDGSAPNAHFMRSNNPAAHYHTCPDSSRHSVIIPYSGPQVGTEYVTEMFAFMCFSSCASGPSRRPVEVIFTLERD
GQTLGRRVVEIRVCACPGRDRKSDERIACGEPSSSQGAKRPRKGSKGPMSKRRRTSSRSQEEEFTVTTKNYHVYEILL
DMKEKLEEKFESWNIQSPDHSMVLSTSEDEGEQPSTSSSPGAVYKRTSLASM

>XP_028403575.1 cellular tumor antigen p53-like [Dendronephthya gigantea]
MESRPDHPSNLIPQEQMFFNNIDPNEAGRTFFANNPGMAMPQDDFMRQPDGNMEECCDPHQYLPPTPSSPLSF
PPVLTSGISNTRHQISTAVTSSDSSCSRPYQQSPYRDNQVSSSFPMNTNGFYQRAQLPTVPTSKQFSGDFNFEISFGP
PTESAPKSATWTYSPKTSKLYTNIGSYCPFQFKTNGKSMSPAFIRAVAMFKGSSNLHDIVKMCANHAENNTDDSQ
MRNNFFIRSNNANAVYLECPQTGRHSVLVPYSGPQAGTNFVTEMFAFKCFSSCASGHGRKPIEIIFTLEDAETGNIL
GRCVVEIRVCACPGRDRRSDEVNLDRVIASNNSNKRASKQKSYNRKKLKKASHEEYTITTTSKEVHDLLVTIKIALENK
LSYKSEFDAVAEEENSSAKRVQPLGASTSHEASIENKQTGYTSCI

>XP_022798783.1 cellular tumor antigen p53-like isoform X1 [Stylophora pistillata]
MRHDARKSDSYLPFDSSSRVEINFGSVKRGLVGDLASRTVQNKMVDNVEPTINTFLQAKQYKSLLDDVREENTSCFL
SPLGTGEDSQDNFLPSDMRPYGQSFPFIPSSQAPVDLPTSTSPNPPAYPHNIPCSPGLVSVHRSSSSISPINTAPYETQ
SSTYSDASTPGTPAASSPNMVPNYMYQPQPQSRLPQIPSTTEYPGDLNFQISFGPITESASKSATWTYSDVCKKLYV
NLNSYCPIKFKTSQAPPHKTYLRAVAVFKGSTNLHDVVKRCPNHMENSNDGSAPNNHFMRSNNPAAGYHTCPESS
RHSIIIPYSGPQVGTEYVTEMFAFMCFSSCASGPSRRPVEVIFTLEREGQTLGRRVVEIRVCACPGRDRKSDERIACGE
PSSSQGAKRTRKGSKGPLSKRRRTSFRSHEEEYSVSTRNYHVYEILQDMKDNLDEKFESWNIQSPEHSTVLSTSEDEG
EQPSTSGSPGALYKRVGQSSM

>XP_020898669.1 cellular tumor antigen p53 isoform X1 [Exaiptasia pallida]
MTNDIVFHHVYSRCHISVILSRLSQKRGSNETPCKHSIIYYSIILFNASHLTMKRKEELEMVDKLMDLSFFNVGGEETD
FNPEIRSMFPGYPIPYGSHGDFPPQTTGPPAYPQNIQGPMYTNVNTAGRRTPVSPVSNQSDMHYDNKSDLSPGHS
PYNSHLPTIPSTTEYPGEMGFAISFGPATESASKSATWTYSESCKKLYVNLASFCPIKFKTDLGPPLGCYLRAVAVFKGS
TNLHDIVKRCPNHSQTSGDSSDNNLHFIRSNNPAAHYHTCPESGRHSVVIPYHGPQVGTEYVTEMFAFMCFSSCPS
GPSRRPVEIIFTLEKDGITLGRRVVEIRVCACPGRDRKSNESLSGVSSTSASSKRKKGQGKSSKKQRATSSGDEGDKE
WTITGKDIRVYNLLMTMKEQLESQFMEETPIHHSEDEDPVFGPSTSTSTATSYPNLRYKRVGHSSM

>XP_004209954.2 PREDICTED: cellular tumor antigen p53-like [Hydra vulgaris]
MVVWNTMESSIRGMIHEPPTPYLNQLMSNHHAEQTSLVLKSEDLNNNTNSVVPFLNGNCSFYGQEQSHYNQVY
PFDYSNGCSININTNLVNTLQTLSSCGANTYARKTEQSLSSREAISYEPNLYLEMQNHSRIIQTNIDHLPDTTNFPGEF
EFQLSLGPHTESAAKSADFTFSSILKKLYVQRASQCPFMFKTEKKPPSNSYVKILPVFKGTHVLSEPVRRCSNHAEVDD
EGGPRYHFIRASNLQSCYNHSDDGRLFVTVPYNGPQVGAEYQIELLSFMCFNSCGHSSHSKRRIDVIFILECEGYVLG
RQVIEVRVCACPGRDRTNEEFSSPNYILTKSCKSRRNSEKKRKSSSNDNNQDYTITTSNPVFYQILRQILVTLETQLPQ
AELILESSTSPISSVSSPDNVS

>THD27310.1 Cellular tumor antigen p53 [Fasciola hepatica]
MFASLTMDREVLSAVREPDRLLFLARQHSHSTGAELSLSIDNTLSTYTDNTVPYLITDPTVESGTTSQTAQDLKQAYL
SLHHLQPQSQAHPIPHRHHHRLHQQTQQKPEQEREQQQSSTDSATGSSNNERARTGLVVTMDLLCAPPHTELSGI
KEPSQGTPIQSATQSTPVSPPSVSSTPSARQSGFNVSPSAMTELDSVGPIRQDSGVDTSMPPKKLYSDDIPVLDSFPG
FYGFDLFRPLCGDTYEAAETKPSTSFFFFKDEQGWTNLYSKKTPAWWTLSYWCQRQPPEGSFLRLTPVYGTSDKQQ
EVIQRCFEDFMAMPTNSLARYSIIVVGNSSADYFYDPNTERLCVTLPYERPKEGCEYSQFNGKFMCFNSCLHNGGQ
GNKKPLFLIVTLERLVAGTEPSKGQCEVLGRQCIKFRSCACPKRDKENSERRTGDSICGSTQGSGKRKKDLERGDRHN
KKWCPQTQDFGATGTKHSFHLNGRWQSPGHELFAHDGSNPLGEDDNDEDSDPRHSRLFVDQDTGSSVIQYNGE
SYHMLLIPTGLPGGLETLAGVRFGLLRTWLYAQSVMMKQSSPSTNRSTNVALKPEVLSENTVTENENRDDPSQTYL
PGRNMEPDTVLHQLLTIESQLAHVIRERIYPTNGCHGPYPYGGESTDSQPNSNGAVVGLSLGSVDETAHANHEFST
AANVGPLSSDSGVSSDAIQHHTPLLMNGNFPSVSLAHRPGLYPTSGSQTSTGYDPDYGNRFQNHLSVGQLYPPHL
MNNTQLRNSIGLSGYFPEITDNYPVLHTIPHSQATSPISDRTGASSNLNSVVVDHADAAKSTTTTTTSAQVRGSRTE

GVTLPTSLSAFTSTVFYPTSYFGSRPTDNSDQTNPNSTSLLDPFSRGHLTTGAAVGMSTSTNGVSTGLCFYPAVGPTT
LSESLLIHEEVISNYLDREKSYDETDGGHQSVLLTRNPIHRRCSSFGSSSPQSPGSSCCDQTTLLEV

>XP_018646355.1 cellular tumor antigen P53, putative [Schistosoma mansoni]
MYGTQHRFKISPNTYHDLENGNSLTTTSIGPRPDTDLDSAFPPKKLYTDDIPVLFLFHHKLYFKDEQGWTNLYAKKTP
TWWTLSYWCQRKPPEGSFIRLTPVYGASDKQQEVIQRCFEDFMAIPTSGMGRYSIVMVGNSLAEYFFDPITERLCV
TLPYETPKEGCEYSQFNGKFMCFNSCLYNGGQGNKKPLFLIITLERLITGSDPSKGHCEVLGRQCIKFRSCACPKRDKE
NNERRTGDSLVIGTPSLSKRRKESSKRERINLLNMYQQTYHFSGDSTDSQPNSTLSTGAMGFNGTGNLDEPVNILN
QDYFNSSNMNGSVGSDLMNSNLPRNLSNHQTNIMQNMAAYILSSTSTNTTTSTTTTATSINNIGNRGFIYNTPNA
VDTNWSYETDSDLRSGQTSHTDQLYESNHQGLIIGNNNNNSTLHVVSELIDPYALVQHLPQSLSSSSPVHQHHHIH
SHHLHSAHVQSQHNHQIHLHQGMNNSTSISDTNDNHSSAFTTTALISDNTMNNKSLTLNTCLLPQITPTTTATVTD
TGTIGTTNFGHDFNTSNFYTSAYFNLTNSNTNADNNSNGSNNNVMDCTNVNQSNDITDQNSTLIDSLSRSSIPSNL
NHRSISQNSTLCNYQLTNHTNLSESLLLSEELLHNCLDNKKKCYDEPDGVHSILTKHLRNTSFCLSSTRSPESCGAEKFV
KLEI

>CDI97221.1 tumor protein p63 [Echinococcus multilocularis]
MSPGGGSGNGSGEMEGKMGFVVDHQPPPKKLFSDDIPVLDPFPGYYGFDLYRPLCEDAYDSIETKPSTAYFFFKDD
QGWTNLYAKKAPTWWTLSYWCQRQPPDGCFIRLTPVYGTSDKQQEVIQRCLEDFMVSPTPGLSRYSMILVGNSN
AEYFYDPTTERLCVTLPYERPKEGCEYSQFNGKFTCFNSCLYNGGQGNKKPLYLIITLERPIKNGSATEGPCEVLGRQC
LKFRSCACPKRDKDNNERRMAQDMGDGNLASNSVLNSKRRREGVSQRSSRGSRGKNFPSTSDRVGTPQFFQQDE
ISSSSGYDDLVQIDGESFHLILMPAYLPGGAHTLRQMRSALLKIFQTARDCEDDRKISNSAASGYSGERQEELLQALN
SMEGRTASLLRDLQTSSTFVNPNSCSTDSQPNSSTSQAFTCPTGGSSGALDPIGSSTLDLLAYDHLHSQHIIKSENSFL
QPFSGQTNEYHRSRMNYPSTLTTTLESAHQQRVSSCFESNTGGTDLPTFNGISMDSSAAAAYAMMPSAFQSFRDS
DSIHHYQPNVFITSSQNTVNSSVPSVVISQDNNGALVAAFGAAVSSSQSSGKLFEEIANSRQQQCHQTSGTDVSMS
NSSCGTASPISPASSNKN

>TGZ61581.1 hypothetical protein CRM22_007927 [Opisthorchis felineus]
MGDTAHPPKKLTSDDIPVLDSFPGYYGFDLYRPLCGDTYEASDTKPSTAFFFFKDEHGWTNLYSKKTPTWWTLSYW
CQRQPPEGSFIRLTPVYGTADKQQEVIQRCFEDFMSIPTSAMSRYSIVLVGNSLADYFFDPVTERLCVTLPYERPKEG
CEYSQFNGKFMCFNSCLYNGGQGNKKPLFLIITLERLVAGTEPSKGQCEVLGRQCIKFRSCACPKRDKENSERRTGEL
SNYHMSGFGKRKKDGDRTDRSGKKGRSHSIDFTTSTAKATRHLTSHWGSPTTESHIHGSNMDDGFEVRHNADQY
GDAEAGQTVINYNGRSYHLLLVPTGLPGGIETLSGVRLGLLRTWLYTSANSIMDKKNSGLISTGPANQTAIKPEVDTD
KETTLPANDSDDVYSSPVNCRASVDSALQQLLVAEMQLAHVLRERFVQNQGPYHHSRDSTDSQPNSDGAVGLGL
GSLDESVNPGQEFSQACAVGPLSSDSGISTDLGQQHHHLSHPQQLGHRNSMGFTQQSHLISDMFSVAPTTHRGS
VLPGTAVNTSSAGSYEVDYASRFSAPSNSHLYTSNQSSEHPADLRSSLGYAYPAELIETYALVQQASSSPGSTISSGHP
SLPAKPQSVSVRNAGNRQNIGSSMSVPESVATALSVNLNHAFTSSAFYPTHYFTARPNGSSDQTSSLLDNFSRNQLA
ASVSGAQSNFCFHPSVGNGALAETLLLPEEVLSSYLERDKGYDEANGVTHTSIGRHPEST

>GAA29806.2 cellular tumor antigen p53 [Clonorchis sinensis]
MERVLSRPGDSDPILAANLHSGLRPLQQSFPDRISQLINGAQNNTVSGGGSEPLSGGTTTSFNVGERARTGLALAM
DLIRMPNVVDKTEHPTSVLSTINRTTTPVCSPPVVSTPGTYTRHSSGFNVSPNGYTDLESAGPVRADAMGDTAHPP
KKLTSDDIPVLDSFPGYYGFDLYRPLCGDTYEASDTKPSTAFFFFKDEHGWTNLYSKKTPTWWTLSYWCQRQPPEG
SFIRLTPVYGTADKQQEVIQRCFEDFMSIPTSAMSRYSIVLVGNSLADYFFDPVTERLCVTLPYERPKEGCEYSQFNGK
FMCFNSCLYNGGQGNKKPLFLIITLERLVAGTEPSKGQCEVLGRQCIKFRSCACPKRDKENSERRTGELGSYHMSGF
GKRKKDGDRTDRSGKKGRSHSIDFTTSIAKSTRHLTSRWGSPTTESHIHGSNMDDGFEVRHNADQYGDAEAGQTV

VNYNGRSYHLLLVPTGLPGGIETLSGVRLGLLRTWLYTSANSIMDKKNGGLINTGPTNQTAIKPEVDADKETTLPAN
DSDDVYSSPVNGRASVDSALQQLLVAEMQLAHVLRERFVQNQGPYHHSRDSTDSQPNSDGAVGLGLGSLDESVN
PGQEFSQACAVGPLSSDSGISTDLGQQHHHLSHPQQLGHRNSMGFTQQSHLISDMFSAAPTTHRGSILPGTAVNT
SSAGSYEVDYASRFSAPSNSHLYTSNQSSEHPADLRSSLGYAYPAELIETYALVQQASASPGSTISSGHPSIPAKPQSVS
VRNAGNRQNIGSSMSVPESVATALSVNLNHAFTSSAFYPTHYFTARPNSSSDQTSSLLDNFSRNQLVASVSGAQSN
FCFHPSVGNGALAETLLLPEEVLSSYLERDKGYDEANGVTHTSIGRHPYRRSPSYSSSPPVSPNSACCEKVVILDM

>VDM22031.1 unnamed protein product, partial [Hydatigera taeniaeformis]
MEALSKLSSGFHDFAAAVSGHTFTHDDVGALPTPPSILPLVPVDTTINSGDGSSVSGPLMNVKMSPGGGSGSGSGE
IEGKMSFVVDHQPPPKKLFSDDIPVLDPFPGYYGFDLYRPLCEDAYDSVETKPSTAYFFFKDDQGWTNLYAKKAPT
WWTLSYWCQRQPPDGCFIRLTPVYGTSDKQQEVIQRFMANQCLEDFMVSPTPGLSRYSMILVGNSNAEYFYDPT
TERLCVTLPYERPKEGCEYSQFNGKFTCFNSCLYNGGQGNKKPLYLIITLERPIKNGSATEGPCEVLGRQCLKFRSCAC
PKRDKDNNERRMAQDLGEGSLSSSSVLSSKRRREGVSQRSSRSSRSKNFPSANDSVGGTPQFFQQDEISSSSGYDD
LVQIDGESFHLILMPAYLPGGA

>RJW71291.1 Cellular tumor antigen p53 [Clonorchis sinensis]
MPPEGSTRAGILPGCSSLDREVERQRSGSNHGPCANLHSGLRPLQQSFPDRISQLINGAQNNTVSGGGSEPLSGGT
TTSFNVGERARTGLALAMDLIRMPNVVDKTEHPTSVLSTINRTTTPVCSPPVVSTPGTYTRHSSGFNVSPNGYTDLE
SAGPVRADAMGDTAHPPKKLTSDDIPVLDSFPGYYGFDLYRPLCGDTYEASDTKPSTAFFFFKDEHGWTNLYSKKTP
TWWTLSYWCQRQPPEGSFIRLTPVYGTADKQQEVIQRCFEDFMSIPTSAMSRYSIVLVGNSLADYFFDPVTERLCVT
LPYERPKEGCEYSQFNGKFMCFNSCLYNGGQGNKKPLFLIITLERLVAGTEPSKGQCEVLGRQCIKFRSCACPKRDKE
NSERRTGELGSYHMSGFGKRKKDGDRTDRSGKKGRSHSIDFTTSIAKSTRHLTSRWGSPTTESHIHGSNMDDGFEV
RHNADQYGDAEAGQTVVNYNGRSYHLLLVPTGLPGGIETLSGVRLGLLRTWLYTSANSIMDKKNGGLINTGPTNQ
TAIKPEVDADKETTLPANDSDGVYSSPVNGRASVDSALQQLLVAEMQLAHVLRERFVQNQGPYHHSRDSTDSQPN
SDGAVGLGLGSLDESVNPGQEFSQACAVGPLSSDSGISTDLGQQHHHLSHPQQLGHRNSMGFTQQSHLISDMFS
AAPTTHRGSILPGTAVNTSSAGSYEVDYASRFSAPSNSHLYTSNQSSEHPADLRSSLGYAYPAELIETYALVQQASASP
GSTISSGHPSIPAKPQSVSVRNAGNRQNIGSSMSVPESVATALSVNLNHAFTSSAFYPTHYFTARPNSSSDQTSSLLD
NFSRNQLVASVSGAQSNFCFHPSVGNGALAETLLLPEEVLSSYLERDKGYDEANGVTHTSIGRHPYRRSPSYSSSPPV
SPNSACCEKVVILDM

>VDD76345.1 unnamed protein product [Mesocestoides corti]
MARQRMTLTLAAVKPAHDSTIFGSQNASTSPALAPRANSSPDWSTASTRPRLPHSTMEALSKLVSPVLSCLVFHPN
GASSSLSLSCTDARLSCGEAILERRRLVPTCWRCSPQLGFGSSVCRPPSSNGHTPLGFHDFAAAAAAVSAPAYHHHH
HHHHDAVGSLPTPPPLLPLATDSVDVSGGGGGALLGGSGLLNVKMSPGGGSGGGGGELEGKMGLPEHQPPPKKL
FSDDIPVLDPFPGYYGFDLYHPLCEDAYDSMETKPSTAYFFFKDDQGWTNLYAKKAPTWWTLSYWCQRQPPEGCL
IRLTPVYGTSDKQQEVIQRCLEDFMTSPTPGISRYSMILVGNSNAEYFYDPLTERLCVTLPYERPKEGCEYSQFNGKFT
CFNSCLYNGGQGNKKPLYLIITLERPMKNGFVVFVSGTATEGPWEVLGRQCLKFRSCACPKRDKDNNERRMAQDL
NDGSLLSGGLPSSKRRRDGASQRAARVNRSKGFTPAGDRIGTPQFFQHEDVSSIAGYDDLVQIDGESFHLILMPAYL
PGGANTLRQIRSALLKVFQTARGGEEERKTHESVASGYSAERHEELLQALNSIEGRTATLLRDLQTSTGFINPNNCSS
DSQPNSSTSQTFPGPNGGSAGAIDLIGSSTLDLLAYDHLHSQHLLKPEGSFLQPFSTQTNDYHRRMSYPSTGATSLD
THQQRVGGCFDGGSGGGDLSAFNGSMESSAAAYATMPSAFQSFRDNGGMHYQPNVFITTSQTTGHSAASVAVS
HDTDGTIVSVFGGGGGGSTASQGGKLFNEVTDGRQQQQQQHQTTATSTATDVSMLESCRLLSAGVQCLILFLLSLN
REFRSLDYNVEVLNSAQRGYLVPGLPIMSRRFTPYRTGTHV

>XP_011405395.1 PREDICTED: cellular tumor antigen p53-like isoform X1 [Amphimedon queenslandica]
MEEPHSDPDSVIHSDEEDLPGSQESLGLSQGTLNELMEAIKSNGSITEEFYVCEEESSSVTENSSSMDSITIQRSLLTPA
SFMNSQQQQQQSQHMLPISSSYSYPQPPPPPLYHVYGDSGSAVSSANTTPTRVTPPGDSPAAMSDCSGSYSHMH
GMPFIRKKVPSNIPMPGEYGFTLILNDDDNSKPPKTVPFTYSNLMKRAYIKRDSTVGMTFSFSKVPPPNAVIRAMAI
HKSPDLIGDILQCCPKHIEDQKKRGHQFPKHFICGAAKTETIYCEDPASGRLSITMPISSLQAKSLTSGSVQAFFVFPCF
TSELHKGPGQVAQLIFTLEIGGVLYGRAVVDIRVCASTGRDRDNDEQKQSTPSTIDPLVDLKKSRKRPALTQNVECAI
PSKVSRTSLSAKQSFTVETYPELFPFVEVCVRMMERVHQVNIESEANLSQLNIGATATDVPDDPLLPSASSQLHGSQ
DIRNVESWLQQLNLSQYKVLLDNGGICDLAQVISLDNNQLKDLGVSNSSHREQLLNGISLINTAATQQPSREDETFT
YSQSTQPYKPQLLSQSSTASGMSNIISPNTLEVLRIQVKKTYSSSSGSESN

>VDP80469.1 unnamed protein product [Echinostoma caproni]
MDREVLSAVSEPDRLLAHQHLHSTGAELSLSNGNSSSASSSSSSSSSSSSASSASTSAAASYADRSLSYLITGTGTVSH
HKPQQQHLPLHSLQTQQTLAHPPPALPLYPQHPPPPPPLPLPSQSTQHHLSQSHHTHHIQQQSHPQPDTDCATGV
STHERARTGLVLTMDLLCASHPDLSGIKEPQGTPVRSATQSTPVSSPSVASTPSARQSGFNVSPSALTELDSVGPMR
QDSGVDTSMPPKKLYSDDIPVLDSFPGYYGFDLFRPLCGDTYEAAETKPSTSFFFFKDEQGWTNLYSKKTPAWWTL
SYWCQTQPPEGSFLRLTPVYGTSDKQQEVIQRCFEDFMAMPTNSLARYSIIVVGNSSADYFYDPNTERLCVTLPYER
PKGEFTSHLTDY

>VDQ00547.1 unnamed protein product [Trichobilharzia regenti]
MDVKFSLVDDDKENSSELDVEEVIQNSSIITKNSLQKVYVTFMRAWPINFVFRPPITPHDFLIRITPMFTYAERCNEV
VQRCKKHEAQTLSEDQALMGKSFVEILGVNCQYMIGNTIPNCPTGQISVCTSVDVLQLLHQSKPEPPSSSVDSDISQ
SLLPEVKGLLHCRLSCYNSCFGSTSRGEVDLIITLEAKSNDSHVGEETYKVFGLSKISVRCCSCPARDAKSDCKQSDSPD
KNSRRLSLNLSSQSNIRLKNDSSSIRRSCPSKIQKVENINQIDSCSSVIIDGKKYYITVVSLI

>XM_001745968.1_Monos
MASQDTLPASQETVPDAETGVFPPMLTRHGSSYKRMSMRDFHEY
LSRINKDGDMASGSMVRRDCMMHKDGWQQVDAEFDVDIDILGGQLPMDTELVDLETPL
PGTGLDDTPQASAKPCGALEDSGEPNPAGFRANLADSSVAAGPGARAIGWTYSPILNT
LFTPMDYSCPIRFATNESVPDLSRIVAHLEYTQTNQRNFVVNRCDMHRQGDSGPFAEH
VLRVNNPQANYHQRQERLAVSVPVASTRSGKVEQNELFEWHCLTSCAGGINRRKIRVV
FRLIDPDQNVLGVQHINVRVCACPVRDRRTHEKAHAKREQANDKRNVIASDPTPSKKP
KLKDHPISMALRKMPSVSTLCQIGSGFDHDQDVQYLAICGRANYEVALKVRDQLNQSA
GLGRVAASSVHLPRSEVTLIKQRVAEATNLDEFLRACNLQSLKRTLENLGYTDLPLIA
TLDAREIEELTLTKQDQRRLKLALVTLNETDKAQDPYLARRRETKLMRVTVRSTPGQA
QAGGAGPTSPFAAAS

>XM_001747604.1_Monos
MEMSLFDDVVDGEVSSSSSSSSSSSSSSSSSSSRFPPLATNMNA
LTMIDWATPILSPLLSFLDSIMTSVVPGSVADGNERGPGQFQVAVETITLQHSDSDLM
VDIPGYGTTAVKRAPMTERDFEMLPSSFRGPYDLQLDLIDENSLKPRNTSPWTVSTPG
LDLTLLQNGSAPIPISSNSLHVPCRWPHFDVLLMIQYSPQLGRFFVHVNADVVLKIVL
ARAPPKGTDLVFRLRYALPEHRKTRVETCVTHQQAGSHFFGAPHNHLMSINREHVTYD
TDSTGHHYARVALDQFPFTDNVYSVPLRFHCFSSCPGSIARRMMQLMVYLEHSEHILG

ITSVDCRCCACPGRDRLSAEKRQQRNSAKRHPAASGTKSKRTRRSGDNMALASSQESN
EGESSQPSEDLSIHILPIHGKTHYRIIKEMIEGMSSARILTPNQTAVTAKANLKLQQR
LREHSQSHSNPPNLELLQRELSTGVSESPSLHKASARAMAIKEEPSAFKADGPTSGNN
SAVEAGDQGSTVPTEMDDQQSGDTEHTPHLDLVPEADALYRNDWKFLQVGTARAHEPGQARTW

>XM_004991339.1_Salp
MQAGARREDSLGRDIKVDIEDLLSTREASTIGISSFDVPFDQLE
LDRTCDFLIETVQLNDGFEDPTPVVDLPGKGRVKLPRVPTTSAELEHTPSKYPGPFNF
QLQFIDDSGGKPVPRATPFTYSPQAQRLFVQMNHKVPFRFSISDRVPNGTWLRIRTRF
TQPEFRNRQD

>XM_004991340.1_Salp
MEVTTLVTHRADGMHAYHLVRCDMPEAVYEKDATGHYSIRIPFD
RCAAQNGTHVFYIELLCFSSCPGGLNRRPFEVTVSLEHSSRILGCDSIDTRVCACPGR
DRGAAEKRLRGDIGKKRGRKKKPPTEPTVSMQDDNKIHTLLVQGRTHYAILHHLVEG
LDATVASSSSPPPSSSSSAAAAVLSSSAARNQAPSADKPLSEASFPAGNDAEETPGGD
TEPESEADDDNDEEEDEQQQLTPRQQVKYAFLNLRLQEAVRAWLSALRLEAYADMFMS
KGYDDLDVVCDLTQDDLDCLGISRPGHRKKLLLAAQKLKSLLHQARAVEAVPDRRLHK
TLSISRTTYRQALPSAD

>XM_004994533.1_Salp
MSGHARLSRQASFANLLRGSSTESISTWISSLGRAPSLTRCSSS
FIRHVTPDASAPLSWAESTTNIEIAASDVPGFSEEFLNLTRSPTRTGLVAAQQQERQQ
QQQQEGEPQHYQQHHHQQQQQQQQQQQQQQQHHEQQSSALTLNLNMEADTRGSAKASS
WMYSGTLHQLFTNIDQVFPVQITSSESVPESAKLHVQLQFSKDPSHPIPRCPTHLAED
TAPHAEHVLQTTMPTADYHMDTSGRAYLVVPIERSKDEGLLCQTLLLKMRCFSSCSGG
MNRRPVDIVFTLINGAQLLAQVSVAVRVCACPARDCRNMERQQAKRAAKSHQGKDVRH
PAKKGKVRGITAICKRQDADLRDSDTFSLNIKGRANYHIIRRIAEALKTNQVNVPWNR
SAGAIVIPDNIHDVASVPEFLARLGLTQYQPLFMGSGFDDLEVLEHLDLNDLSAIGVE
DGIHQDTLLYAAAKLARSQQHHGEGCRPRGRCTSLHSPDVVDTHDHNNSTISISSNCH
VFPDTSQQMQYTTQPHFPPMQLAPSNGVPHSLPSAAPSVAHAAKPSSGPGTSTSSAVA
TTAPVQQQQQQQQQQQQQQQQQQQQQQQQQQHSGPLIHLPVPSSSSA

>Caps
MDMVRLTRGQSFQQQQQQQKSASQQNASGDGAAAAGAGAGAGAGAAAGADADADSKGSHDPADLNSTLLL
GDSQFGETTGTSLLNMSGLRGFNTPSSSSSHHSHHSHHSNSSNSSFLGRPRTLPVQKPLSILQGSAPEPF
VEPLTIGEPIYASPNSLGEAGFLLSVDVSNARHSAISSAYSYSEALGTLFTNFDVGVPFVFRVAKPPPTV
HPLHIRATLRYKQMQFMKEPVRRCPLHLSIDSDLHLLRACDQDTVYSVDYHGRASIAVPFTPTMQPLVPV
LINTLKDAHVPLVTRHPSSTHGEASRSNPYVCWSMTWFLKFMCYTSCTGGMNRRATEIVFTLEDSQGLIY
GAQALDFRTCASPSRDRKQLEKSPPPIPVGSIIPPEVVTAHQGSALADPGTTASTPTRRSAQPVVPSPVV
AASPTPAPGLLSSGRKAGAIPVTGVTPRPLVVIPAASSAQTAPTAPAAAIAAPSPALTEAHAAPTTLLRD
RSITAIAKRRHADDDDEIFTISIRGRDNFVLVSKIVEGLNALAQRTSAAVASKLERTTTPPVVEDDPSAP
PALSALDTLAQAAAGHVRAPDEAAPATHERKRARLADDDTKAGRATEASGESISTWLGSFGLKQYAVNFA
KNGYTNTPQILNLCEDDLKSIVRVKAHRVRIAAAIDELADAQAEPTSTPPMSSDGDVTVVRMTIRRTTSS
TAK

>Sphae
MYNLPLQDHAPAAEPSTSDPQLNIQPELTLPPPRQTPPVLDSSHTPMPGIYNFRVRLGHFDSTNPTGKLT
GSTTYSSLLDRLFINMGVYTPITLFSHTPPPEGAVVRCFVVFSSQEKCTTTVTRCPNHRSATDHSNTGND
PQKAPLTHILQVSHPQAQYLTTPSGVESAVVPYNARESSVKNSAAKKERTAGFGAFQDTTSIRYMCYSSC
AGSINRENIHTVFRLELGSDILGVASLDTKICSCPSRDRTKAEKLTRQQEASGTTGSSRKRSEKGRDSPS
HSTDIASTGATKDSHTSERIQAPSIVHPHSSQATLVGKRSQRLIQNVQHKLPTRINTADTHTAHPMRWST
AQSTRIATADSTVGIDEDAVYYVAVRGRQNYEVLSRVADGLNALAQRPPRARSFRQHNPLPTRTPSLLVT
SNPPPPPTTTANTAAPTNTNTNTNTNTSTDTKTVFSRPGQNSTIPSLSSRRNPSLVSSTTSVPPAQPRTA
ASSRSLRTVPSRERRVSGTVPSDVPVSESPARNTR

>Creol
MTESESADMTGKLGQPSMSFSQIFNSFAENGSTVVGEHGNEVGQEVMFSVMRRSTSNAQATMATRDYNTNAQ
QTFQPDGQATNAGYYASEPLEDNGLYSGRIEIEDGGYQYIMNPMNNPLGILPATLPSYLDNVSASYDAHGSLANLIG
LPFVNDGRAADSNAQGIQSHPELVPPAAQSPPLPLDSSHTPVPGIYGFGVNVGHLDTSNPTGKLNGSTTYSKLLDRL
FININVYTPITIATNKQPPPGAMVRCFVVFSSQEKCRDIVTRCPNHRSPTDPTNQSVDANEVPLSHMLHVSHPKALY
CPSNTGIESVLIPYRPTEISFAKASKIKRPSGTTDSTTDAPTLQEHTYVRFMCFSSCPGSINRENIHAVFRLELGDEILGV
ASLDTKICSCPSRDKAKAEKLARAHVGMSDRNSNAKSMKHEVRGSDGAGTVKRRSPITHSVSSASTPSNQKGFVGP
ASVLTRSASQRLQENNMQTRTKGQADSSMYKSNIHSHPNGININGSTKLMARGREDSNRLGSGVDNGLEHTNGG
GSTRTYYNVSAMLNSNTREPGGHNKFDDELYYVTVRGRKNYEILSRVAEGLTCTSQKPQRALSYRRHPGAPAKVQSI
VVSNHSSKEPVKPISSERARATAIPRVVTSSITSPHIPTSQGDITTPKRSKRLLVSSEAKIQSPARRAIGKGVDITVNGSE
VRQNNPKKSRVNGQNKPATRQGNKTHRAVQNNADGSSTEEEPELADRSIIPPLTSKSRISVPTRSHQTRRSSARLN

>Ichth
MHNLHNLLPRQNTSALGTKTKDNESNRTGRENQLQHDTLELSLERMDVDIDLSNNMFGLPSFPNSGLTNY
DLTSTTNSVVDMLEYLDPILTPSPVLDPSWALPQAQHQAQPQSQQLPLPMLTRSQSNARFEEPNFEGPYH
FNISFVTKTESGGKSDAKGATWLYGSQRDLLYVTMNTTVPINLTIEGNPPADANVRVKVVYANPEDYMER
VVRCPTHADRKEQATKPFPDQIIRVDHLAATNELDRLNHYSVLIPYSKLKSRPQLRLRYLCYSSCVASIN
KRPVSTVIILEDASGRLLGQRSVSTKICACPGRDLKKDNPITAEKDKWKNNRNVKGQQTNDIDPHPSRTT
FVNPKNEILQSTKSIVQDNNNMYTVNRTIRGRANFEMINKIIDSLNLMDNLQNDSINPNHSSVDPPALHR
QFNLRDMCPTLALIPPPIIQNHKTKEQRMVSREKTPTIESDEEEIVRDTKPTKKFKQGSPFSPPGPSLQK
TNSFSRMQ

>Chromos
MSFPNQNRVPGSEQTWLENLVMRFPSEGGGNEFMLNNRHHHHPNKENEMLDEVEFKFEAVNCFSAVPDTF
QAGGSSNSNMSGVGDKDSSCHSDSVDRYIQGHLQPVGNHQSNSNNNNRCRAPLVPTNSLGRESTSSNTTA
PEILGRNYVRGALSYEFPHNVKGESGAHDRKLSLDAGRTAAMDSASRSSPLLQSPKAELSSLVDENALQH
PPETSNISTCPSERGFARLEQVLDDVAVDGTSAQHSDVYDGNAQVREQIPFDVCFEGSSLQSKATTWGYS
STLNKVFLNLDTLCPIPLSIEQENIPRGFILRATARFTKPQHYNETVVRCPNHEQKDPNFKSSHKDKIVP
TSHVVRCENPSAVYETDAASGHHSVYIPIHTRTKSGSSDLSSFADDSKANDNGNDDKPKKTNIIVPFKFM
CFSSCPGGINRRPFELQLTIENNGVVFATRSVGIRVCACPLRDKRSEELTFMQKNKRLLVDCEKEEESTG
AHGSDEKSSKWLVSKRRKTNIPSSLSGNSLASLSKTVPSTIDKHGNEIFNIKVKGRQNYDIVARVVEGLN
ALYGDITHGRQKNAEKKAIGSKPGDYSGTGTDTAEESDEEESEMSGRKGQEDKLMTSYLEVLSKYSPEAL
KCMPVREFLKAVNLDTYYQSFKDNGYDDIQLVTCIDKSDLDILNISLGGHRKKLVAAARALFSRLKGPVE
TTRRRGSIVYTQTTKRGSFTMKKTYSATSSQPSGSIFPADFPNIISSLGGKDDGSL

>Corall
MSFQQRGPMKAPPPPLDLQGSGANRPELMRQQSQDGAERAITALAQVAFQESVALVEGRIQFVDRNQVLN
QQTNGGPGNANGEEAKPQSTEPSKDSSALLLDVPMADIGLLLNKEQEEELKKEVKRKVTYFDVDIPTHKP
FGAFSKKLEKLFALPHVPYLFSFYTPITEGSIQIKLKFKNPYYQHEPVERCEQCSKKEEPQPDAPKHFVT
CTADERGESNAEHTITDDGPIVTMRFNLQREVAPRVNTGSGNVHCVQRNLKFGCLNSHLGGKNKRDLEIN
LSLLSTEGVQLASKTIDLKVCKRPQRDADLEEQGEAMESVDAMGMYGNVPQGQRPPQEEKDKSKEDWHLI
KVRDFEDWKLLNQMAEALSFFREHVHNAYSQKLLSGQKGEDQDIGSKRGPPDADGPAAKLRKLSPEENQR
TLYEWLERNGLKQLQPVLASNNVHSFDDLWSICASEQVPFLPGDVKQKMKTILEKDNPRADAVRDAFENA
TRLLLKMEITKVFDLNDSQGNAFKGLQRNAVPTPAAPLSAILPSPARFTTQNADWLLQAGSLDELNVRFA
NPFSPLITPGLQAELLSGQNHFFGQNSLDMIHQQQQTNERLGIGLNPTHVQQHQEQPTPDIFF
